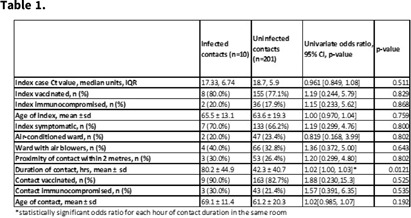# Duration of exposure is the most important risk factor for nosocomial COVID-19 in open multibed wards

**DOI:** 10.1017/ash.2022.130

**Published:** 2022-05-16

**Authors:** Hwang Ching Chan, Alicia Ang, Nazira Fauzi, Revathi Sridhar, Annie Poh, Isaac Low, Dale Fisher, Paul Tambyah, Jyoti Somani

## Abstract

**Background:** The National University Hospital (NUH) is a 1,200 bed tertiary-care hospital with no documented nosocomial transmission of COVID-19 among patients for the first year and a half of the pandemic, despite 65% of the patients being housed in 4- to 8-bedded open cubicles with shared bathrooms. However, this arrangement changed in late September 2021 with large community clusters including in healthcare institutions nationally associated with the spread of the δ (delta) variant of SARS-CoV-2. We conducted a retrospective review of hospital epidemiology data to determine risk factors for SARS-COV-2 transmission during this period. **Methods:** Index patients were defined as the first patient in an open cubicle with a confirmed positive SARS-CoV-2 PCR test. Contacts were defined as being in the same cubicle as a patient before isolation from 2 days before symptom onset, up to 7 days from positive test if asymptomatic. Clinical and patient movement data were obtained manually from routine clinical records. Proximity of the contact from the index was classified as within, or more than, 2 m away, according to the prevailing definition from the Singapore Ministry of Health. A univariate analysis was performed to identify risk factors for nosocomial acquisition of SARS-CoV-2. The analysis was deemed exempt from ethics review (reference no. NHG-DSRB-2021/01026). **Results:** From October 1 to November 30, 2021, 30 index cases occurred in open cubicles identified (median, 9 days after admission; IQR, 19 days). Contact tracing yielded 211 contacts, of whom 10 (4.7%) were infected. Linear regression analysis found the duration of contact for each hour spent in the same room as the index case was the only statistically significant risk variable for contracting COVID-19, with an odds ratio 1.02 (Table 1). **Conclusions:** Patients in open cubicles are at risk for nosocomial transmission of COVID-19 and other infections. The duration of contact appeared to be more important than vaccination status of index or ward ventilation status. Larger multicentered studies are needed to validate this finding, which has significant implications for infection prevention strategies and pandemic planning.

**Funding:** None

**Disclosures:** None

**Table:**